# Can endoscopists judge a book by its cover when it comes to Barrett cancer?

**DOI:** 10.1002/ueg2.12638

**Published:** 2024-07-15

**Authors:** V. Bos, R. E. Pouw

**Affiliations:** ^1^ Department of Gastroenterology and Hepatology Amsterdam UMC location University of Amsterdam Amsterdam The Netherlands; ^2^ Amsterdam Gastroenterology Endocrinology Metabolism Amsterdam The Netherlands; ^3^ Cancer Center Amsterdam Amsterdam the Netherlands

**Keywords:** adenocarcinoma, biopsy, diagnosis, endoscopy, ESD, ESGE, esophageal cancer, GERD, Paris, treatment

Guidelines strongly recommend endoscopic resection (ER) to treat Barrett esophagus (BE) associated lesions, based on high‐quality evidence.^1^ Endoscopic submucosal dissection (ESD) is advised over band‐assisted ER for lesions with suspicion on submucosal invasion, Paris 0‐Is/0‐IIc lesions, malignant lesions >2 cm or lesions with signs of fibrosis.^2^ This recommendation, however, is a weak recommendation based on low quality evidence.

The recommendation is rational with regard to the Paris type 0‐Is lesions, since the bulky part of such lesions would fill up the band‐assisted ER‐cap, prohibiting deep resection, a problem overcome by ESD. Also, in case of scarring, ESD allows for the best chance to dissect through the fibrotic tissue in a controlled way. The recommendation is less convincing when it comes to the 2 cm cut‐off, which is because band‐assisted ER would require piecemeal resection for lesions >2 cm, whereas en‐bloc resection with ESD is not size restricted. There are, however, no high‐quality studies providing evidence that en‐bloc resection of Barrett cancer results in higher eradication rates, or lower recurrence rates. Yet, ESD has not been around as long as band‐assisted ER, so more high quality data may be coming from randomized trials that are currently underway (NCT05276791; NCT03427346).

For lesions with a suspicion of submucosal invasion, which is the case for Paris type 0‐Is/0‐IIc lesions, the European Society of Gastrointestinal Endoscopy (ESGE) guideline advises ESD to increase the chances of en‐bloc and deep submucosal resection.^1^ One may debate this argument, since ESD does not always result in adequate submucosal resection as demonstrated in a qualitative analysis of submucosal excision depth in ER for early BE cancer. This study showed that no submucosa was present in about a third of the resection area, for both band‐assisted ER and ESD.^3^

Nonetheless, expert‐opinion is that en‐bloc resection with ESD might result in an oncological more adequate resection, with a tissue specimen that allows for optimal histological staging. Especially since indications for curative endoscopic management of Barrett cancer are expanding from low‐risk T1a lesions to T1b lesions,^1^, ^2^, ^4^ ESD is advised as the preferred technique for lesions with suspicion of submucosal invasion.

But can endoscopists judge a book by its cover when it comes to Barrett cancer? Younis et al. conducted an interesting study to answer this question.^5^ The authors assessed the positive and negative predictive value of nine Barrett experts to correctly stage T1 cancer (T1a or T1b, and Sm1 or Sm2/3), and assessed agreement on infiltration depth, Paris classification, and subsequently advised management strategy (i.e. band‐assisted ER, ESD, or surgery). Participants were shown 3 ‐ 4 images of 202 retrospectively collected early Barrett cancer cases, combined with demographics (i.e. age, sex, BE length) and pre‐ER biopsy results. Results showed that the experts could not reliably stage T1 cancer, and that agreement on infiltration depth, Paris classification, and recommended management strategy was only low to moderate. The study had some limitations. The distribution of T1a/T1b cases was unequal, and a substantial portion (27%) of ER's performed during the inclusion period were excluded because of inclusion in a randomized controlled trial, introducing a potentially significant selection bias. However, the results could be seen in line with previously reported moderate inter‐observer agreement on the Paris classification and questions its usefulness in staging of lesions and determining ER techniques.^6^

Despite the sobering outcomes, a mere solace for the expert endoscopists from the study may be that even for expert pathologists assessment of submucosal is a challenge. A Dutch study amongst 13 dedicated Barrett pathologists from expert centers demonstrated a discrepancy of up to 45% when differentiating T1a versus T1b cancers.^7^ In the study by Younis et al. initial histology was assessed by expert gastrointestinal pathologists, without reassessment for this study.^5^ One could question, if histological revision would have changed outcomes, or would have only complicated analysis by introducing more inter‐observer variation.

The difficulties and challenges in assessment of Barrett cancer and deciding on the best treatment may not be easy to overcome. Attempts to improve assessment of infiltration depth using artificial intelligence have also been disappointing, with similar accuracy rates as expert endoscopists (60%–70%), in line with current study's accuracy rates.^8^, ^9^

We can only echo the authors that precise endoscopic distinction between mucosal and submucosal involvement of BE cancer by experts as a basis for choosing the resection technique has limited predictive values and high inter‐observer variability, even amongst experts.

While awaiting more data from prospective studies comparing band‐assisted ER and ESD, we may need to accept that assessment of submucosal invasion in Barrett cancer and choosing the “right” ER technique is a relative and not an absolute truth.

## CONFLICT OF INTEREST STATEMENT

R. E. Pouw is consultant for MicroTech Europe and Medtronic BV and has received speaker fee from Pentax and Boston Scientific. V. Bos has no disclosures.

## Data Availability

Data sharing not applicable to this article as no datasets were generated or analyzed during the current study.
